# Dietary factors and postmenopausal bleeding: A Mendelian randomization investigation

**DOI:** 10.1097/MD.0000000000041650

**Published:** 2025-05-30

**Authors:** Jiali Ren, Wenwen Yang, Tian Tian

**Affiliations:** a Department of Obstetrics and Gynecology, Enshi Tujia and Miao Autonomous Prefecture Central Hospital, Enshi, Hubei, China; b The First Clinical Medical College, Lanzhou University, Lanzhou, Gansu, China.

**Keywords:** dietary factors, FinnGen biobank, Mendelian randomization, postmenopausal bleeding, UK biobank

## Abstract

Understanding modifiable risk factors for postmenopausal bleeding (PMB) is crucial for developing preventive strategies. However, few studies have explored the relationships between dietary factors and PMB. This study employed Mendelian randomization (MR) to investigate the causal relationships between 20 dietary factors (including vegetables, fruits, meats, and beverages) and PMB risk via data from the UK Biobank and FinnGen Biobank. 3 MR analysis methods were utilized: inverse-variance-weighted, weighted median, and MR-Egger. The inverse-variance-weighted analysis identified that poultry intake (odds ratio [OR] = 3.00, *P* = .0394), nonoily fish intake (OR = 0.372, *P* = .0145), and coffee intake (OR = 0.508, *P* < .0001) were associated with PMB. However, the weighted median method only confirmed the association with coffee intake, and the MR-Egger method did not support any associations. The remaining 17 dietary factors showed no significant association with PMB. This study suggests that poultry intake may increase the risk of PMB, whereas nonoily fish and coffee intake may have protective effects. These findings provide new insights into the dietary determinants of PMB, underscoring the need for further research to elucidate the underlying mechanisms and confirm these associations in diverse populations.

## 1. Introduction

Approximately 10% of postmenopausal women experience postmenopausal bleeding (PMB).^[1]^ It is considered a clinical symptom with multiple potential underlying causes, ranging from benign conditions such as endometrial polyps and atrophy to more severe issues such as endometrial hyperplasia or cancer.^[2,3]^ As such, PMB serves as an important clinical sign of endometrial abnormalities, including malignancy.^[4]^ Understanding the modifiable risk factors for PMB is crucial, as it provides opportunities for early prevention and management strategies. Despite the high prevalence of PMB, there is a significant gap in the literature regarding the dietary factors that may influence its occurrence. While numerous studies have investigated the role of diet in the health of women in general,^[5–7]^ few have focused on how specific dietary components might affect PMB risk. This study employs Mendelian randomization (MR), a method that uses genetic variants as instrumental variables (IVs) to mitigate the biases that often affect observational studies, to explore the causal relationships between various dietary factors and PMB. MR, a method that leverages genetic variants as IVs, provides a robust framework for mitigating confounding biases and inferring causal effects from observational data.^[8,9]^ The foundation of the MR study includes the independence assumption (confounding factors do not influence the IVs), the relevance assumption (IVs are strongly associated with the exposure), and the exclusion restriction assumption (IVs affect the outcome only through the exposure). By leveraging large-scale biobank data from the UK Biobank and FinnGen Biobank, this research aims to clarify the influence of 20 dietary factors, including vegetables, fruits, meats, and beverages, on the risk of PMB. Understanding these associations is essential for developing targeted dietary recommendations that could reduce the incidence of PMB and improve the quality of life for postmenopausal women. The dietary factors considered in this study are diverse and include common items such as vegetables, fruits, meat, alcohol, coffee, and fish, each of which may have distinct effects on hormonal regulation,^[5]^ inflammation,^[10]^ and endometrial health.^[6]^ Given the complex nature of PMB and the limited evidence linking diet to its occurrence, this study seeks to provide novel insights into how specific dietary components might influence the risk of PMB. The findings of this study could have significant implications for both clinical practice and public health strategies aimed at reducing PMB-related morbidity.

## 2. Method

### 2.1. Data sources

We utilized 20 food categories as exposures, primarily sourced from the UK Biobank, including vegetables and fruits, meat, bread and cereal, cheese, salt, alcohol, coffee, tea, and water, in our investigation. Table 1 provides an overview of these exposures, while Table S1, Supplemental Digital Content, https://links.lww.com/MD/P2 details the specific measurement methods for each dietary category. The outcome of this research, PMB, was obtained directly from the FinnGen Biobank, which includes 111,583 controls of European descent and 12,600 cases of European descent. PMB was primarily defined via the International Classification of Diseases, 10th Revision code (N95.0)^[11]^ for cause of death and hospital discharge data. Since the study data are publicly available, anonymized, and do not involve human subjects directly, an ethical review is deemed unnecessary.

**Table 1:** Information on exposure and outcome datasets.

IEU GWAS id	Exposure or outcome	Participants included in analysis
ieu-b-73	Alcoholic drinks per week	335,394 European-descent individuals
ukb-b-5779	Alcohol intake frequency	462,346 European-descent individuals
ukb-b-6324	Processed meat intake	461,981 European-descent individuals
ukb-b-8006	Poultry intake	461,900 European-descent individuals
ukb-b-2862	Beef intake	461,053 European-descent individuals
ukb-b-17627	Nonoily fish intake	460,880 European-descent individuals
ukb-b-2209	Oily fish intake	460,443 European-descent individuals
ukb-b-5640	Pork intake	460,162 European-descent individuals
ukb-b-14179	Lamb/mutton intake	460,006 European-descent individuals
ukb-b-11348	Bread intake	452,236 European-descent individuals
ukb-b-1489	Cheese intake	451,486 European-descent individuals
ukb-b-8089	Cooked vegetable intake	448,651 European-descent individuals
ukb-b-6066	Tea intake	447,485 European-descent individuals
ukb-b-3881	Fresh fruit intake	446,462 European-descent individuals
ukb-b-15926	Cereal intake	441,640 European-descent individuals
ukb-b-1996	Salad/ raw vegetable intake	435,435 European-descent individuals
ukb-b-5237	Coffee intake	428,860 European-descent individuals
ukb-b-16576	Dried fruit intake	421,764 European-descent individuals
ukb-b-8121	Salt added to food	462,630 European-descent individuals
ukb-b-14898	Water intake	427,588 European-descent individuals
N/A	Postmenopausal bleeding	12,600 European-descent cases and 111,583 European-descent controls

We present the IDs and sample sizes of 20 diet-related factors from the IEU OpenGWAS project. More information about exposure and outcomes is available at the IEU OpenGWAS project (https://gwas.mrcieu.ac.uk/) and the FinnGen Biobank (https://r10.risteys.finngen.fi/endpoints/N14_POSTMENBLEED).

GWAS = genome-wide association studies, IEU = integrative epidemiology unit, NA = not applicable.

### 2.2. Selection of IVs

IVs, such as single-nucleotide polymorphisms (SNPs), are used in MR studies to minimize confounding and infer possible causal links between exposures and outcomes. We applied several criteria to select SNPs for our investigation, including a clumping window of 10,000 kb, considerable linkage disequilibrium (*r*^2^ < 0.001), and significance below the genome-wide threshold (*P* < 5 × 10^–8^). To reduce bias and in accordance with MR assumptions, SNPs exhibiting notable associations with the outcome (*P* < 5 × 10^–8^) were removed from each analysis.

### 2.3. Statistical analysis

Three well-known MR analysis methods were employed in our research to assess causal linkages: the inverse-variance-weighted (IVW) method was chosen as the primary method due to its superior capacity to detect causal relationships compared to the weighted median (WM) and MR-Egger methods. To guarantee the reliability of the outcomes, the IVW approach needs to fulfill specific requirements: the need for horizontal pleiotropy to stay negligible or balanced.^[12]^ Applying false discovery rate corrections^[13]^ to the *P* values derived from the IVW method in this study helps reduce the risk of false positives in multiple hypothesis testing. To find outliers, we utilized the MR pleiotropy residual sum and outlier method. We employed Cochran Q test to quantify heterogeneity. The fixed-effects IVW approach was adopted when there was no significant heterogeneity (*P* > .05); otherwise, the random-effects IVW method was adopted. For all the statistical analyses, R (version 4.3.2) with the TwoSampleMR package^[14]^ (version 0.5.10) was employed.

## 3. Results

This study assessed the impact of 20 diet-related factors on PMB by applying the MR analysis method. With more than 330,000 participants for each dietary factor in our study, an in-depth investigation of the impact of diet on PMB was possible. The primary focus of our research is on the analytical results from the IVW method, which is the most effective of the 3 approaches for determining causal linkages.

We investigated the effects of 20 dietary components on PMB after excluding SNPs associated with PMB, and the results of the MR pleiotropy residual sum and outlier method, the 3MR analysis techniques, and horizontal pleiotropy are presented in Table S2, Supplemental Digital Content, https://links.lww.com/MD/P3 and Table 2.

**Table 2:** The results of the IVW method on the influence of dietary factors on postmenopausal bleeding.

Outcome	Exposure	Number of SNPs	Results of the analysis using the IVW method					Results of the analysis using the weighted_median method				Results of the analysis using the MR_Egger method				Horizontal pleiotropy		
			IVW_OR	IVW_lci95	IVW_uci95	IVW_pval	IVW_q_value	Weighted_median_OR	Weighted_median_lci95	Weighted_median_uci95	Weighted_median_pval	MR_Egger_OR	MR_Egger_lci95	MR_Egger_uci95	MR_Egger_pval	egger_intercept	se	pval
Postmenopausal bleeding	Alcoholic drinks per week	28	1.43E + 00	9.33E−01	2.20E + 00	9.98E−02	3.22E−01	1.19E + 00	6.79E−01	2.08E + 00	5.45E−01	1.15E + 00	4.34E−01	3.05E + 00	7.82E−01	4.33E−03	8.68E−03	6.22E−01
Postmenopausal bleeding	Alcohol intake frequency	87	9.28E−01	7.82E−01	1.10E + 00	3.89E−01	5.99E−01	9.19E−01	7.31E−01	1.16E + 00	4.69E−01	8.70E−01	5.05E−01	1.50E + 00	6.16E−01	1.60E−03	6.50E−03	8.07E−01
Postmenopausal bleeding	Processed meat intake	21	6.63E−01	3.58E−01	1.23E + 00	1.92E−01	4.27E−01	5.06E−01	2.40E−01	1.07E + 00	7.32E−02	4.71E−01	2.12E−02	1.04E + 01	6.39E−01	5.21E−03	2.36E−02	8.27E−01
Postmenopausal bleeding	Poultry intake	7	3.00E + 00	1.06E + 00	8.55E + 00	3.94E−02	2.56E−01	2.33E + 00	5.65E−01	9.65E + 00	2.42E−01	3.91E−04	8.97E−18	1.70E + 10	6.45E−01	9.69E−02	1.73E−01	6.00E−01
Postmenopausal bleeding	Beef intake	12	9.34E−01	3.43E−01	2.54E + 00	8.93E−01	9.86E−01	6.02E−01	1.97E−01	1.84E + 00	3.74E−01	4.14E−01	8.54E−04	2.01E + 02	7.86E−01	1.01E−02	3.87E−02	7.99E−01
Postmenopausal bleeding	Nonoily fish intake	11	3.72E−01	1.69E−01	8.22E−01	1.45E−02	1.45E−01	4.14E−01	1.37E−01	1.25E + 00	1.18E−01	2.46E + 00	5.63E−02	1.07E + 02	6.52E−01	−2.35E−02	2.34E−02	3.43E−01
Postmenopausal bleeding	Oily fish intake	52	9.89E−01	6.85E−01	1.43E + 00	9.52E−01	9.86E−01	9.07E−01	5.64E−01	1.46E + 00	6.88E−01	8.48E−01	1.81E−01	3.99E + 00	8.36E−01	2.28E−03	1.14E−02	8.43E−01
Postmenopausal bleeding	Pork intake	10	9.92E−01	3.78E−01	2.60E + 00	9.86E−01	9.86E−01	8.82E−01	2.51E−01	3.10E + 00	8.45E−01	1.87E−01	5.00E−04	6.95E + 01	5.94E−01	1.68E−02	3.00E−02	5.90E−01
Postmenopausal bleeding	Lamb/mutton intake	26	1.76E + 00	8.75E−01	3.54E + 00	1.13E−01	3.22E−01	1.61E + 00	6.85E−01	3.77E + 00	2.76E−01	2.87E + 00	1.36E−01	6.07E + 01	5.05E−01	−5.46E−03	1.70E−02	7.50E−01
Postmenopausal bleeding	Bread intake	25	9.25E−01	5.35E−01	1.60E + 00	7.81E−01	9.86E−01	1.26E + 00	6.63E−01	2.39E + 00	4.81E−01	8.89E−01	6.45E−02	1.23E + 01	9.31E−01	5.79E−04	1.90E−02	9.76E−01
Postmenopausal bleeding	Cheese intake	53	8.56E−01	6.51E−01	1.13E + 00	2.67E−01	4.85E−01	8.15E−01	5.38E−01	1.24E + 00	3.35E−01	1.73E + 00	5.37E−01	5.55E + 00	3.63E−01	−1.20E−02	9.87E−03	2.31E−01
Postmenopausal bleeding	Cooked vegetable intake	15	8.24E−01	3.27E−01	2.08E + 00	6.82E−01	9.74E−01	9.39E−01	3.05E−01	2.89E + 00	9.12E−01	4.13E−04	2.08E−08	8.21E + 00	1.47E−01	7.89E−02	5.22E−02	1.55E−01
Postmenopausal bleeding	Tea intake	33	7.00E−01	4.89E−01	1.00E + 00	5.11E−02	2.56E−01	5.87E−01	3.76E−01	9.16E−01	1.90E−02	3.70E−01	1.78E−01	7.68E−01	1.20E−02	1.43E−02	7.38E−03	6.15E−02
Postmenopausal bleeding	Fresh fruit intake	49	7.46E−01	4.25E−01	1.31E + 00	3.05E−01	5.09E−01	1.04E + 00	4.64E−01	2.31E + 00	9.32E−01	1.73E + 00	2.58E−01	1.16E + 01	5.75E−01	−8.08E−03	8.90E−03	3.68E−01
Postmenopausal bleeding	Cereal intake	34	1.03E + 00	6.24E−01	1.70E + 00	9.11E−01	9.86E−01	1.25E + 00	6.83E−01	2.30E + 00	4.67E−01	7.07E−01	8.48E−02	5.89E + 00	7.50E−01	5.49E−03	1.53E−02	7.23E−01
Postmenopausal bleeding	Salad/ raw vegetable intake	12	9.64E−01	3.59E−01	2.59E + 00	9.42E−01	9.86E−01	9.94E−01	2.67E−01	3.70E + 00	9.93E−01	4.59E−01	4.78E−03	4.41E + 01	7.45E−01	7.91E−03	2.43E−02	7.51E−01
Postmenopausal bleeding	Coffee intake	35	5.08E−01	3.63E−01	7.10E−01	7.33E−05	1.47E−03	5.09E−01	3.16E−01	8.21E−01	5.62E−03	5.58E−01	2.86E−01	1.09E + 00	9.69E−02	−1.80E−03	5.61E−03	7.51E−01
Postmenopausal bleeding	Dried fruit intake	33	6.53E−01	3.89E−01	1.09E + 00	1.06E−01	3.22E−01	6.31E−01	3.21E−01	1.24E + 00	1.82E−01	2.96E−01	2.62E−02	3.35E + 00	3.33E−01	9.68E−03	1.48E−02	5.18E−01
Postmenopausal bleeding	Salt added to food	86	1.26E + 00	9.23E−01	1.72E + 00	1.47E−01	3.67E−01	1.71E + 00	1.17E + 00	2.52E + 00	6.06E−03	1.39E + 00	5.03E−01	3.83E + 00	5.28E−01	−1.47E−03	7.36E−03	8.43E−01
Postmenopausal bleeding	Water intake	34	1.30E + 00	8.28E−01	2.05E + 00	2.52E−01	4.85E−01	1.71E + 00	9.48E−01	3.09E + 00	7.48E−02	3.50E + 00	1.02E + 00	1.21E + 01	5.58E−02	−1.57E−02	9.39E−03	1.04E−01

We analyzed the relationship between 20 dietary factors and postmenopausal bleeding. The IVW method, known for its heightened sensitivity in detecting causality, serves as our primary tool to ascertain the presence of a causal relationship. The MR-Egger method is also employed for detecting horizontal pleiotropy due to its allowance for the presence of nonzero intercepts (The columns in the table corresponding to “egger_intercept,” “se,” and “pval”), a pval below 0.05 suggests the existence of horizontal pleiotropy. q_value: The *P* value post FDR method (Benjamini and Hochberg) corrected; SNPs: Single-nucleotide polymorphisms; pval:*P*-value; IVW: Inverse Variance Weighted method; lci95:Lower 95% Confidence Interval; uci95:Upper 95% confidence interval; OR: odds ratio.

The IVW method revealed that among the 20 dietary factors, 3 were associated with PMB: poultry intake (odds ratio [OR] = 3.00, *P* = .0394), nonoily fish (OR = 0.372, *P* = .0145), and coffee (OR = 0.508, *P* < .0001).

The remaining 17 dietary factors, included weekly alcohol consumption (OR = 1.43, *P* = .0998), alcohol intake frequency (OR = 0.928, *P* = .389), salt added to food (OR = 1.26, *P* = .147), processed meat consumption(OR = 0.663, *P* = .192), beef consumption (OR = 0.934, *P* = .893), oily fish consumption (OR = 0.989, *P* = .952), pork consumption (OR = 0.992, *P* = .986), mutton consumption (OR = 1.76, *P* = .113), bread consumption (OR = 0.925, *P* = .781), cheese consumption (OR = 0.856, *P* = .267), cooked vegetable consumption (OR = 0.824, *P* = .682), tea consumption (OR = 0.700, *P* = .0511), fresh fruit consumption (OR = 0.746, *P* = .305), cereal consumption (OR = 1.03, *P* = .911), raw vegetable consumption (OR = 0.964, *P* = .942), dried fruit consumption (OR = 0.653, *P* = .106), and water consumption (OR = 1.30, *P* = .252), were not associated with PMB.

Among the 3 factors identified by the IVW method, only coffee intake remained associated with PMB in the WM method. However, none of these 3 factors were associated with PMB in the MR-Egger method. Further analysis results can be found in Table 2.

## 4. Discussion

This study, which assessed the impact of 20 diet-related factors on PMB, demonstrated that different dietary patterns have varying effects on this condition. Among the 20 diet-related factors, only poultry intake was identified as a risk factor for PMB, whereas nonoily fish intake and coffee intake were identified as protective factors.

The finding that poultry intake increases the risk of PMB is intriguing and warrants further investigation. Poultry, a common source of protein and various nutrients,^[15,16]^ has not been extensively studied in the context of PMB. There are currently very few studies investigating the relationship between poultry intake and PMB. Our research indicates that excessive poultry consumption may increase the risk of PMB, highlighting the significance of our study in elucidating the connection between poultry intake and PMB. Possible mechanisms could involve certain fats or additives used in poultry processing that might influence hormonal pathways or endometrial health. However, further biochemical and clinical studies are needed to explore these potential mechanisms.

A study has shown that coffee consumption may be associated with a reduced risk of endometrial cancer in postmenopausal women.^[17]^ Endometrial cancer is one of the most common causes of PMB. A significant portion of patients with PMB are eventually diagnosed with endometrial cancer.^[18]^ Another study found that habitual coffee consumption is associated with a reduced risk of PMB.^[19]^ Our study indicated that higher coffee consumption significantly reduced the risk of PMB. Coffee, which is widely consumed for its stimulant properties, has various health benefits, including anti-inflammatory and antioxidant effects.^[20–22]^ These physiological functions of coffee may contribute to the prevention and treatment of certain chronic diseases.^[23]^ These properties might play a role in maintaining endometrial health and preventing abnormal bleeding. Nonoily fish, which are rich in lean protein and essential nutrients, may contribute to hormonal balance and reduce inflammation, thus lowering the risk of PMB.

The discrepancies observed among the different MR methods highlight the complexity of establishing causal relationships in dietary studies. While the IVW method identified 3 dietary factors associated with PMB, the WM method confirmed only the association with coffee intake, and the MR-Egger method did not support any associations. This variation underscores the importance of using multiple analytical approaches and interpreting results with caution. The potential influence of horizontal pleiotropy, where genetic variants affect the outcome through pathways other than exposure, remains a critical concern in MR studies. Moreover, the lack of association for the remaining 17 dietary factors suggests that these foods may not have a significant impact on PMB or that their effects are too subtle to detect with the current sample size and methods. It is also possible that these dietary factors interact with each other or with other lifestyle factors in complex ways that are not captured by our analysis.

Unquestionably, this research has certain intrinsic limitations. Firstly, our inability to further divide the dietary components in our study limits our research potential and complicates the investigation of complex interactions and minute variations among different food ingredients. Secondly, stratified analyses are not possible due to the lack of summary-level genome-wide association studies data that are age-categorized, which restricts our capacity to look at genetic associations between various age groups. Thirdly, since the exposure and outcome data were predominantly collected from European populations, the applicability of our findings to other populations remains uncertain.

## 5. Conclusion

This study provides novel insights into the dietary factors associated with PMB, identifying poultry intake as a potential risk factor and nonoily fish and coffee intake as potential protective factors. These findings contribute to our understanding of the influence of diet on PMB and underscore the need for further research to elucidate the underlying mechanisms and confirm these associations in diverse populations.

## Acknowledgments

Special thanks to the UK Biobank, the FinnGen Biobank, and the IEU open GWAS project developed by The MRC Integrative Epidemiology Unit (IEU) at the University of Bristol.

## Author contributions

**Conceptualization:** Jiali Ren, Wenwen Yang, Tian Tian.

**Data curation:** Jiali Ren, Wenwen Yang, Tian Tian.

**Formal analysis:** Jiali Ren, Tian Tian.

**Funding acquisition:** Tian Tian.

**Investigation:** Tian Tian.

**Methodology:** Tian Tian.

**Project administration:** Tian Tian.

**Resources:** Jiali Ren, Tian Tian.

**Software:** Wenwen Yang, Tian Tian.

**Supervision:** Tian Tian.

**Validation:** Tian Tian.

**Visualization:** Tian Tian.

**Writing – original draft:** Wenwen Yang, Tian Tian.

**Writing – review & editing:** Wenwen Yang, Tian Tian.

## Supplementary Material




