# Supplementary material for: Dietary factors and postmenopausal bleeding: A Mendelian randomization investigation
Source: Medicine (Baltimore). 2025 May 30;104(22):e41650. doi: 10.1097/MD.0000000000041650 (PMC12129501; doi:10.1097/MD.0000000000041650)
Supplement: Supplementary file 1 [file medi-104-e41650-s001.pdf]

Supplementary Table 1. Information on exposure and outcome datasets.

IEU GWAS id	Exposure or outcome	definition	Source
ieu-b-73	Alcoholic drinks per week	1. Defined as the average number of drinks a participant reported drinking each week, aggregated across all types of alcohol. If a study recorded binned response ranges (e.g., 1-4 drinks per week, 5-10 drinks per week) we used the midpoint of the range. For example, if an individual reported 1-5 drinks per week, we assume they drank 2.5 drinks per week on average. 2. This was measured in a variety of ways. a. In the past week, how many alcoholic beverages did you have? b. Thinking about the past year, on the average how many drinks did you have each week? 3. This phenotype was left-anchored at 1 and log-transformed prior to analysis, in order to prevent outliers from having undue leverage on analyses.	N/A
ukb-b-5779	Alcohol intake frequency	ACE touchscreen question "About how often do you drink alcohol?" If the participant activated the Help button they were shown the message: If this varies a lot, please provide an average considering your intake over the last year. Options include: (1)Daily or almost daily (2)Three or four times a week (3)Once or twice a week (4)One to three times a month (5)Special occasions only (6)Never (7)Prefer not to answer	https://biobank.ctsu.ox.ac.uk/crystal/field.cgi?id=1558
ukb-b-6324	Processed meat intake	ACE touchscreen question "How often do you eat processed meats (such as bacon, ham, sausages, meat pies, kebabs, burgers, chicken nuggets)?" If the participant activated the Help button they were shown the message: Please provide an average considering your intake over the last year. If you are unsure, please provide an estimate or select Do not know. Options include:(1)never (2)once a week (3)less than once a week (4)2-4 times a week (5)5-6 time a week (6)once or more daily (7)do not know (8)prefer not to answer.	https://biobank.ctsu.ox.ac.uk/crystal/field.cgi?id=1349
ukb-b-8006	Poultry intake	ACE touchscreen question "How often do you eat chicken, turkey or other poultry? (Do not count processed meats)" If the participant activated the Help button they were shown the message: Please provide an average considering your intake over the last year. If you are unsure, please provide an estimate or select Do not know. Poultry include fowl (e.g. chicken, turkey, quail), waterfowl (e.g. duck, goose) and game (e.g. pheasant). Options include:(1)never (2)once a week (3)less than once a week (4)2-4 times a week (5)5-6 time a week (6)once or more daily (7)do not know (8)prefer not to answer.	https://biobank.ctsu.ox.ac.uk/crystal/field.cgi?id=1359
ukb-b-2862	Beef intake	ACE touchscreen question "How often do you eat beef? (Do not count processed meats)" If the participant activated the Help button they were shown the message: Please provide an average considering your intake over the last year.If you are unsure, please provide an estimate or select Do not know. Options include:(1)never (2)once a week (3)less than once a week (4)2-4 times a week (5)5-6 time a week (6)once or more daily (7)do not know (8)prefer not to answer.	https://biobank.ctsu.ox.ac.uk/crystal/field.cgi?id=1369
ukb-b-17627	Non-oily fish intake	ACE touchscreen question "How often do you eat other types of fish? (e.g. cod, tinned tuna, haddock)" If the participant activated the Help button they were shown the message: Please provide an average considering your intake over the last year.If you are unsure, please provide an estimate or select Do not know. Options include:(1)never (2)once a week (3)less than once a week (4)2-4 times a week (5)5-6 time a week (6)once or more daily (7)do not know (8)prefer not to answer.	https://biobank.ctsu.ox.ac.uk/crystal/field.cgi?id=1339
ukb-b-2209	Oily fish intake	ACE touchscreen question "How often do you eat oily fish? (e.g. sardines, salmon, mackerel, herring)" If the participant activated the Help button they were shown the message: Please provide an average considering your intake over the last year.If you are unsure, please provide an estimate or select Do not know. Oily fish include:Salmon Anchovies, Trout Swordfish, Mackerel Bloater, Herring Cacha, Sardines Carp, Pilchards Hilsa, Kipper Jack fish, Eel Katla, Whitebait Orange roughy, Tuna (fresh only) Pangas, Sprats. Options include:(1)never (2)once a week (3)less than once a week (4)2-4 times a week (5)5-6 time a week (6)once or more daily (7)do not know (8)prefer not to answer.	https://biobank.ctsu.ox.ac.uk/crystal/field.cgi?id=1329
ukb-b-5640	Pork intake	ACE touchscreen question "How often do you eat pork? (Do not count processed meats such as bacon or ham)" If the participant activated the Help button they were shown the message: Please provide an average considering your intake over the last year. If you are unsure, please provide an estimate or select Do not know. Options include:(1)never (2)once a week (3)less than once a week (4)2-4 times a	https://biobank.ctsu.ox.ac.uk/crystal/field.cgi?id=1389

		week (5)5-6 time a week (6)once or more daily (7)do not know (8)prefer not to answer.	
ukb-b-14179	Lamb/mutton intake	ACE touchscreen question "How often do you eat lamb/mutton? (Do not count processed meats)"<br>If the participant activated the Help button they were shown the message: Please provide an average considering your intake over the last year. If you are unsure, please provide an estimate or select Do not know.<br>Options include:(1)never (2)once a week (3)less than once a week (4)2-4 times a week (5)5-6 time a week (6)once or more daily (7)do not know (8)prefer not to answer.	https://biobank.ctsu.ox.ac.uk/crystal/field.cgi?id=1379
ukb-b-11348	Bread intake	ACE touchscreen question "How many slices of bread do you eat each WEEK?"<br>The following checks were performed:<br>If answer < 0 then rejected<br>If answer > 250 then rejected<br>If answer > 50 then participant asked to confirm<br>If the participant activated the Help button they were shown the message:<br>For other types of bread:<br>- one bread roll = 2 slices<br>- one pitta bread = 2 slices	https://biobank.ctsu.ox.ac.uk/crystal/field.cgi?id=1438
ukb-b-1489	Cheese intake	ACE touchscreen question "How often do you eat cheese? (Include cheese in pizzas, quiches, cheese sauce etc)"<br>If the participant activated the Help button they were shown the message: Please provide an average considering your intake over the last year. If you are unsure, please provide an estimate or select Do not know.<br>Options include:(1)never (2)once a week (3)less than once a week (4)2-4 times a week (5)5-6 time a week (6)once or more daily (7)do not know (8)prefer not to answer.	https://biobank.ctsu.ox.ac.uk/crystal/field.cgi?id=1408
ukb-b-8089	Cooked vegetable intake	ACE touchscreen question "On average how many heaped tablespoons of COOKED vegetables would you eat per DAY? (Do not include potatoes; put '0' if you do not eat any)"<br>The following checks were performed: If answer > 50 then rejected<br>If the participant activated the Help button they were shown the message:Please provide an average considering your intake over the last year. If you are unsure, please provide an estimate or select Do not know. If you have less than one tablespoon a day select Less than one.	https://biobank.ctsu.ox.ac.uk/crystal/field.cgi?id=1289
ukb-b-6066	Tea intake	ACE touchscreen question "How many cups of tea do you drink each DAY? (Include black and green tea)"<br>The following checks were performed:<br><br>If answer < 0 then rejected<br>If answer > 99 then rejected<br>If answer > 20 then participant asked to confirm<br>If the participant activated the Help button they were shown the message: Please provide an average considering your intake over the last year. If you are unsure, please provide an estimate or select Do not know.	https://biobank.ctsu.ox.ac.uk/crystal/field.cgi?id=1488
ukb-b-3881	Fresh fruit intake	ACE touchscreen question "About how many pieces of FRESH fruit would you eat per DAY? (Count one apple, one banana, 10 grapes etc as one piece; put '0' if you do not eat any)"<br>The following checks were performed:<br><br>If answer > 50 then rejected<br>If the participant activated the Help button they were shown the message:<br><br>Please provide an average considering your intake over the last year.<br>If you are unsure, please provide an estimate or select Do not know.	https://biobank.ctsu.ox.ac.uk/crystal/field.cgi?id=1309
ukb-b-15926	Cereal intake	ACE touchscreen question "How many bowls of cereal do you eat a WEEK?"<br>The following checks were performed:<br><br>If answer < 0 then rejected<br>If answer > 99 then rejected<br>If answer > 14 then participant asked to confirm<br>If the participant activated the Help button they were shown the message:<br><br>Please provide an average considering your intake over the last year.<br>If you are unsure, please provide an estimate or select Do not know.	https://biobank.ctsu.ox.ac.uk/crystal/field.cgi?id=1458

ukb-b-1996	Salad / raw vegetable intake	ACE touchscreen question "On average how many heaped tablespoons of SALAD or RAW vegetables would you eat per DAY? (Include lettuce, tomato in sandwiches; put '0' if you do not eat any)"<br>The following checks were performed:<br><br>If answer > 50 then rejected<br>If the participant activated the Help button they were shown the message:<br><br>Please provide an average considering your intake over the last year.<br>If you are unsure, please provide an estimate or select Do not know.<br>If you have less than one tablespoon a day select Less than one.	https://biobank.ctsu.ox.ac.uk/crystal/field.cgi?id=1299
ukb-b-5237	Coffee intake	ACE touchscreen question "How many cups of coffee do you drink each DAY? (Include decaffeinated coffee)"<br>The following checks were performed:<br><br>If answer < 0 then rejected<br>If answer > 99 then rejected<br>If answer > 10 then participant asked to confirm<br>If the participant activated the Help button they were shown the message:<br><br>Please provide an average considering your intake over the last year.<br>If you are unsure, please provide an estimate or select Do not know.	https://biobank.ctsu.ox.ac.uk/crystal/field.cgi?id=1498
ukb-b-16576	Dried fruit intake	ACE touchscreen question "About how many pieces of DRIED fruit would you eat per DAY? (Count one prune, one dried apricot, 10 raisins as one piece; put '0' if you do not eat any)"<br>The following checks were performed:<br><br>If answer > 100 then rejected<br>If the participant activated the Help button they were shown the message:<br><br>Please provide an average considering your intake over the last year.<br>If you are unsure, please provide an estimate or select Do not know.	https://biobank.ctsu.ox.ac.uk/crystal/field.cgi?id=1319
ukb-b-8121	Salt added to food	ACE touchscreen question "Do you add salt to your food? (Do not include salt used in cooking)"<br>If the participant activated the Help button they were shown the message:<br><br>Please provide an average considering your intake over the last year<br>If you are unsure, please provide an estimate or select Do not know.	https://biobank.ctsu.ox.ac.uk/crystal/field.cgi?id=1478
ukb-b-14898	Water intake	ACE touchscreen question "How many glasses of water do you drink each DAY? "<br>The following checks were performed:<br><br>If answer < 0 then rejected<br>If answer > 99 then rejected<br>If answer > 10 then participant asked to confirm<br>If the participant activated the Help button they were shown the message:<br><br>Please provide an average considering your intake over the last year.<br>If you are unsure, please provide an estimate or select Do not know.	https://biobank.ctsu.ox.ac.uk/crystal/field.cgi?id=1528
N/A	Postmenopausal bleeding	The Endpoint definition for postmenopausal bleeding is in the Finngen biobank	https://r10.risteys.finngen.fi/endpoints/N14_POSTMENBLEED

Information on exposure and outcome datasets. The data used in our study were mainly processed by the MRC Integrative Epidemiology Unit (IEU) at the University of Bristol from the UK Biobank. The data of the UK Biobank and the FinnGen biobank may also be partially updated. Therefore, the data from their official website and the data from IEU may not be completely consistent. More information on exposures and outcome can be obtained on the website provided in the table.
