# Supplementary material for: Dietary factors and postmenopausal bleeding: A Mendelian randomization investigation
Source: Medicine (Baltimore). 2025 May 30;104(22):e41650. doi: 10.1097/MD.0000000000041650 (PMC12129501; doi:10.1097/MD.0000000000041650)
Supplement: Supplementary file 2 [file medi-104-e41650-s002.pdf]

Supplementary Table 2. The results of the three Mendelian randomization methods on the influence of dietary factors on postmenopausal bleeding.

outcome	exposure	Number of SNPs	IVW_Beta	IVW_se	IVW_pval	IVW_OR	IVW_lci95	IVW_uci95	IVW_q_value	Weighted_median_Beta	Weighted_median_se	Weighted_median_pval	Weighted_median_OR	Weighted_median_lci95	Weighted_median_uci95	MR_Egger_Beta	MR_Egger_se	MR_Egger_pval	MR_Egger_OR	MR_Egger_lci95	MR_Egger_uci95	Q	Q_pval	egger_intercept	se	pval	Raw_Causal.Estimate	Raw_Sd	Raw_P_value	Outlier-corrected_Causal.Estimate	Outlier-corrected_Sd	Outlier-corrected_P_value
Postmenopausal bleeding || id:N14_POSTMENBLEED	Alcoholic drinks per week || id:ieu-b-73	28	0.360838087	0.219223514	0.099767344	1.434531173	0.933476023	2.204534059	0.322003244	0.172526212	0.285180315	0.545197354	1.188302968	0.679480185	2.078153231	0.138911978	0.497249378	0.782178238	1.149022956	0.433572261	3.045060466	37.05882203	0.093974754	0.00433058	0.008679126	0.621999655	0.360838087	0.219223514	0.111360227	NA	NA	NA
Postmenopausal bleeding || id:N14_POSTMENBLEED	Alcohol intake frequency. || id:ukb-b-5779	87	-0.074982286	0.087134807	0.389495875	0.927759921	0.78210482	1.100541064	0.599224423	-0.084702747	0.116882262	0.468645694	0.918785356	0.730671007	1.155330542	-0.139693755	0.277776632	0.616335477	0.869624517	0.504525978	1.498925396	117.3603448	0.013919835	0.001595807	0.00650004	0.806665944	-0.074982286	0.087134807	0.391888602	NA	NA	NA
Postmenopausal bleeding || id:N14_POSTMENBLEED	Processed meat intake || id:ukb-b-6324	21	-0.410499487	0.314604169	0.191956664	0.663318848	0.358035312	1.22890642	0.426570365	-0.681449563	0.380356406	0.073195788	0.505883151	0.240040381	1.06614463	-0.752729645	1.581196681	0.639466467	0.471078918	0.021239871	10.44805514	35.27288604	0.01869814	0.005214856	0.023588021	0.827388111	-0.410499487	0.314604169	0.206771371	NA	NA	NA
Postmenopausal bleeding || id:N14_POSTMENBLEED	Poultry intake || id:ukb-b-8006	7	1.099975648	0.533863472	0.039359718	3.004092866	1.055065914	8.553564122	0.255672849	0.84750055	0.723999572	0.241764263	2.333817875	0.564658778	9.646012926	-7.847204159	16.02308363	0.645044965	0.000390843	8.97E-18	17026550537	2.904110464	0.82078222	0.096907389	0.173450531	0.600459716	1.099975648	0.371416461	0.025231566	NA	NA	NA
Postmenopausal bleeding || id:N14_POSTMENBLEED	Beef intake || id:ukb-b-2862	12	-0.068599957	0.510382346	0.893079202	0.933700126	0.343369087	2.538947034	0.986482818	-0.50705857	0.570513174	0.374123332	0.602264495	0.19685962	1.842544048	-0.880763406	3.15556784	0.785844588	0.414466385	0.000853917	201.1698469	18.99288242	0.061221392	0.010106112	0.038700938	0.799288703	-0.068599957	0.510382346	0.89550694	NA	NA	NA
Postmenopausal bleeding || id:N14_POSTMENBLEED	Non-oily fish intake || id:ukb-b-17627	11	-0.987586148	0.404168826	0.014545607	0.372474706	0.168679123	0.822493048	0.145456074	-0.88149072	0.56434872	0.118297165	0.414165047	0.137021941	1.251862912	0.898859456	1.926463447	0.651884567	2.456799425	0.056302919	107.2033838	10.16317692	0.426295832	-0.023470038	0.023434666	0.342745215	-0.987586148	0.404168826	0.034640978	NA	NA	NA
Postmenopausal bleeding || id:N14_POSTMENBLEED	Oily fish intake || id:ukb-b-2209	52	-0.011352368	0.187164316	0.951634328	0.988711827	0.685097286	1.426879215	0.986482818	-0.097521283	0.242840618	0.687989068	0.907083036	0.563555818	1.460014444	-0.164419729	0.789407685	0.835854602	0.848385855	0.180566023	3.986124014	73.62228267	0.020748811	0.002275281	0.011393111	0.842519926	-0.011352368	0.187164316	0.951871397	NA	NA	NA
Postmenopausal bleeding || id:N14_POSTMENBLEED	Pork intake || id:ukb-b-5640	10	-0.00834699	0.492677833	0.986482818	0.99168775	0.377571418	2.604658473	0.986482818	-0.125287793	0.641298813	0.845106665	0.882242963	0.251017288	3.100792986	-1.679266679	3.021001429	0.593504601	0.186510677	0.000500236	69.53957474	7.02552036	0.634462254	0.016807886	0.029981605	0.590416349	-0.00834699	0.435292331	0.985119412	NA	NA	NA
Postmenopausal bleeding || id:N14_POSTMENBLEED	Lamb/mutton intake || id:ukb-b-14179	26	0.565897911	0.356770406	0.112701135	1.76102832	0.875139405	3.543687697	0.322003244	0.4735552	0.435006003	0.276322543	1.605692617	0.684506892	3.766578264	1.053332572	1.55719334	0.505234912	2.867190333	0.135502339	60.66891872	35.08999862	0.08658731	-0.005457904	0.016954916	0.750310339	0.565897911	0.356770406	0.125270239	NA	NA	NA
Postmenopausal bleeding || id:N14_POSTMENBLEED	Bread intake || id:ukb-b-11348	25	-0.077666254	0.279842693	0.781369028	0.925273182	0.534641991	1.601315412	0.986482818	0.231048519	0.327658485	0.480716631	1.259920368	0.662879462	2.394702844	-0.117511458	1.338387597	0.930795015	0.889130324	0.064521863	12.25247836	38.77647481	0.028820582	0.000579223	0.019006961	0.975951738	-0.077666254	0.279842693	0.78374572	-0.238127924	0.255330942	0.360701551
Postmenopausal bleeding || id:N14_POSTMENBLEED	Cheese intake || id:ukb-b-1489	53	-0.155125639	0.139666363	0.266703021	0.856307585	0.651243471	1.125942466	0.484914583	-0.204207507	0.211922607	0.335249152	0.815293175	0.538172673	1.23511095	0.546549297	0.595716523	0.363217316	1.727282383	0.537377158	5.55197479	52.44592278	0.456606388	-0.011960176	0.009873521	0.231348766	-0.155125639	0.139666363	0.271812537	NA	NA	NA
Postmenopausal bleeding || id:N14_POSTMENBLEED	Cooked vegetable intake || id:ukb-b-8089	15	-0.193422227	0.472208908	0.682090917	0.824133929	0.326622017	2.079457898	0.974415595	-0.063365177	0.573419575	0.91200965	0.938600656	0.305053672	2.887921934	-7.792007916	5.049954671	0.146819643	0.000413023	2.08E-08	8.214519333	21.89034372	0.080895764	0.078916084	0.052236479	0.154772998	-0.193422227	0.472208908	0.688289341	NA	NA	NA
Postmenopausal bleeding || id:N14_POSTMENBLEED	Tea intake || id:ukb-b-6066	33	-0.357101189	0.183096072	0.05113457	0.699701692	0.488718075	1.001768674	0.255672849	-0.532315893	0.226895493	0.01897183	0.587243401	0.376427031	0.916126589	-0.994171302	0.372413086	0.011980767	0.370029961	0.17833331	0.767787978	52.09697102	0.013855143	0.014315061	0.007378718	0.061518072	-0.357101189	0.183096072	0.059939973	NA	NA	NA
Postmenopausal bleeding || id:N14_POSTMENBLEED	Fresh fruit intake || id:ukb-b-3881	49	-0.293548616	0.286411322	0.305401062	0.745612973	0.425319423	1.307108669	0.509001771	0.035082743	0.410046092	0.931817749	1.035705403	0.463658218	2.313526732	0.549051735	0.971302391	0.574576201	1.731610214	0.258024445	11.62089095	65.41786814	0.047913933	-0.008078039	0.008896313	0.368499631	-0.293548616	0.286411322	0.310538709	NA	NA	NA
Postmenopausal bleeding || id:N14_POSTMENBLEED	Cereal intake || id:ukb-b-15926	34	0.028531841	0.255530431	0.911095129	1.028942773	0.623561549	1.69786484	0.986482818	0.22489547	0.30951388	0.467465714	1.252191817	0.682664513	2.296859317	-0.347248092	1.081710298	0.750284269	0.706629997	0.084805042	5.88792764	55.21812482	0.009007321	0.005492159	0.01534981	0.722842484	0.028531841	0.255530431	0.911771529	NA	NA	NA
Postmenopausal bleeding || id:N14_POSTMENBLEED	Salad / raw vegetable intake || id:ukb-b-1996	12	-0.036552546	0.503747322	0.942155247	0.964107433	0.35919233	2.587758882	0.986482818	-0.005636597	0.670624276	0.993293859	0.994379258	0.267119226	3.701680792	-0.778181628	2.32920794	0.745207084	0.459240322	0.004779453	44.12673942	8.914621986	0.629772962	0.007909292	0.024252525	0.751053965	-0.036552546	0.453490166	0.937205692	NA	NA	NA
Postmenopausal bleeding || id:N14_POSTMENBLEED	Coffee intake || id:ukb-b-5237	35	-0.678152962	0.171016606	7.33E-05	0.507553598	0.363002517	0.709666304	0.001465326	-0.675186991	0.243827466	0.00562083	0.509061222	0.315660243	0.820956498	-0.583668622	0.341589977	0.096900825	0.557848074	0.285593737	1.089640399	33.56802328	0.488658223	-0.001796965	0.005610529	0.750772011	-0.678152962	0.169926733	0.00033242	NA	NA	NA
Postmenopausal bleeding || id:N14_POSTMENBLEED	Dried fruit intake || id:ukb-b-16576	33	-0.426572368	0.26368668	0.105722011	0.652742626	0.389302633	1.094451718	0.322003244	-0.460759563	0.345303766	0.182085874	0.630804328	0.320601954	1.241146834	-1.216302268	1.237434725	0.333255251	0.29632387	0.026208426	3.350366671	42.64056982	0.09899143	0.00967746	0.014808996	0.51826164	-0.426572368	0.26368668	0.115538468	NA	NA	NA
Postmenopausal bleeding || id:N14_POSTMENBLEED	Salt added to food || id:ukb-b-8121	86	0.229785266	0.158384129	0.146832794	1.258329775	0.922518706	1.716381264	0.367081985	0.538393529	0.196178329	0.006062	1.71325236	1.166355587	2.51658558	0.328058665	0.518274749	0.528464803	1.388270412	0.502700938	3.833879337	142.3120771	9.81E-05	-0.001466471	0.007359559	0.842540394	0.229785266	0.158384129	0.150513281	NA	NA	NA
Postmenopausal bleeding || id:N14_POSTMENBLEED	Water intake || id:ukb-b-14898	34	0.265324073	0.23149041	0.251730698	1.303853451	0.828285705	2.05247273	0.484914583	0.537402377	0.301613675	0.074788813	1.711555109	0.947659114	3.091217979	1.253912565	0.631813164	0.055816065	3.5040259	1.01568043	12.0886424	49.40236617	0.033159506	-0.015719905	0.009385571	0.103700759	0.265324073	0.23149041	0.259974126	NA	NA	NA

The results of the three Mendelian randomization methods on the influence of dietary factors on postmenopausal bleeding.We present the outcomes of three Mendelian randomization methods, including Beta and OR values for each of the three methods. Note that there exists a conversion relationship between them (Beta=In(OR)), introducing some redundancy. The IVW method, known for its heightened sensitivity in detecting causality, serves as our primary tool to ascertain the presence of a causal relationship. The MR-Egger method is also employed for detecting horizontal pleiotropy due to its allowance for the presence of non-zero intercepts (The columns in the table corresponding to "egger_intercept", "se", and "pval"), a p_value below 0.05 suggests the existence of horizontal pleiotropy. Heterogeneity was assessed using Cochran's Q test (The columns in the table corresponding to "Q" and "Q_pval"）. To evaluate the impact of outliers on result significance, we applied the MR-PRESSO method (The columns in the table corresponding to "Raw_Causal.Estimate", "Raw_Sd", "Raw_P_value", "Outlier-corrected_Causal.Estimate", "Outlier-corrected_Sd", and "Outlier-corrected_P_value"; Raw represents the outcomes of the analysis encompassing all SNPs, while Outlier-corrected signifies the results excluding outliers. The MR-PRESSO method not only serves to detect the presence of outliers but also provides a means to assess causality. Through this approach, it is possible to observe whether the presence of outliers affects the significance of the results. IVW: Inverse Variance Weighted method;OR: odds ratio; Weighted_median: Weighted median method; MR_Egger: MR-Egger method; SNPs: Single-nucleotide polymorphisms; lci95:Lower 95% Confidence Interval; uci95:Upper 95% confidence interval; q_value: The P value post FDR method (Benjamini and Hochberg) corrected; NA: not applicable; pval:p-value; NA indicates the absence of outliers.
